# Using ancestry-informative markers to identify fine structure across 15 populations of European origin

**DOI:** 10.1038/ejhg.2014.1

**Published:** 2014-02-19

**Authors:** Laura M Huckins, Vesna Boraska, Christopher S Franklin, James A B Floyd, Lorraine Southam, GCAN, V Boraska, C S Franklin, J A B Floyd, L M Thornton, L M Huckins, L Southam, N William Rayner, I Tachmazidou, K L Klump, J Treasure, C M Lewis, U Schmidt, F Tozzi, K Kiezebrink, J Hebebrand, P Gorwood, R A H Adan, M J H Kas, A F avaro, P Santonastaso, F Fernández-Aranda, M Gratacos, F Rybakowski, M Dmitrzak-Weglarz, J Kaprio, A Keski-Rahkonen, A Raevuori, E F Van Furth, M C T Slof-Op t Landt, J I Hudson, T Reichborn-Kjennerud, G P S Knudsen, P Monteleone, A S Kaplan, A Karwautz, H Hakonarson, W H Berrettini, Y Guo, D Li, N J Schork, G Komaki, T Ando, H Inoko, T Esko, K Fischer, K Männik, A Metspalu, J H Baker, R D Cone, J Dackor, J E DeSocio, C E Hilliard, J K O'Toole, J Pantel, J P Szatkiewicz, C Taico, S Zerwas, S E Trace, O S P Davis, S Helder, K Bühren, R Burghardt, M de Zwaan, K Egberts, S Ehrlich, B Herpertz-Dahlmann, W Herzog, H Imgart, A Scherag, S Scherag, S Zipfel, C Boni, N Ramoz, A Versini, M K Brandys, U N Danner, C de Kove, J Hendriks, B P C Koeleman, R A Ophoff, E Strengman, A A van Elburg, A Bruson, M Clementi, D Degortes, M Forzan, E Tenconi, E Docampo, G Escaramís, S Jiménez-Murcia, J Lissowska, A Rajewski, N Szeszenia-Dabrowska, A Slopien, J Hauser, L Karhunen, I Meulenbelt, P E Slagboom, A Tortorella, M Maj, G Dedoussis, D Dikeos, F Gonidakis, K Tziouvas, A Tsitsika, H Papezova, L Slachtova, D Martaskova, J L Kennedy, R D Levitan, Z Yilmaz, J Huemer, D Koubek, E Merl, G Wagner, P Lichtenstein, G Breen, S Cohen-Woods, A Farmer, P McGuffin, S Cichon, I Giegling, S Herms, D Rujescu, S Schreiber, H-E Wichmann, C Dina, R Sladek, G Gambaro, N Soranzo, A Julia, S Marsal, R a Rabionet, V Gaborieau, D M Dick, A Palotie, S Ripatti, E Widén, O A Andreassen, T Espeseth, A Lundervold, I Reinvang, V M Steen, S Le Hellard, M Mattingsda, I Ntalla, V Bencko, L Foretova, V Janout, M Navratilova, S Gallinger, D Pinto, S W Scherer, H Aschauer, L Carlberg, A Schosser, L Alfredsson, B Ding, L Klareskog, L Padyukov, C Finan, G Kalsi, M Roberts, D W Logan, L Peltonen, G R S Ritchie, P Courtet, S Guillame, I Jaussent, J C Barrett, X Estivill, A Hinney, P F Sullivan, D A Collier, E Zeggini, C M Bulik, WTCCC3, Carl A Anderson, Jeffrey C Barrett, James AB Floyd, Christopher S Franklin, Ralph McGinnis, Nicole Soranzo, Eleftheria Zeggini, Jennifer Sambrook, Jonathan Stephens, Willem H Ouwehand, Wendy L McArdle, Susan M Ring, David P Strachan, Graeme Alexander, Cynthia M Bulik, David A Collier, Peter J Conlon, Anna Dominiczak, Audrey Duncanson, Adrian Hill, Cordelia Langford, Graham Lord, Alexander P Maxwell, Linda Morgan, Leena Peltonen, Richard N Sandford, Neil Sheerin, Nicole Soranzo, Fredrik O Vannberg, Jeffrey C Barrett, D N A Genotyping, Hannah Blackburn, Wei-Min Chen, Sarah Edkins, Mathew Gillman, Emma Gray, Sarah E Hunt, Cordelia Langford, Suna nengut-Gumuscu, Simon Potter, Stephen S Rich, Douglas Simpkin, Pamela Whittaker, Patrick F Sullivan, Cynthia M Bulik, David A Collier, Chris Tyler-Smith, Eleftheria Zeggini, Ioanna Tachmazidou

**Affiliations:** 1 The Wellcome Trust Sanger Institute (WTSI), Hinxton, UK; 2 University of Split School of Medicine, Split, Croatia; 3 University of North Carolina, Chapel Hill, NC, USA; 4 King's College, London, UK; 5 GCAN members are listed before the references.; 6 WTCCC3 members are listed before the references.

**Keywords:** population stratification, AIMs, principal component analysis

## Abstract

The Wellcome Trust Case Control Consortium 3 anorexia nervosa genome-wide association scan includes 2907 cases from 15 different populations of European origin genotyped on the Illumina 670K chip. We compared methods for identifying population stratification, and suggest list of markers that may help to counter this problem. It is usual to identify population structure in such studies using only common variants with minor allele frequency (MAF) >5% we find that this may result in highly informative SNPs being discarded, and suggest that instead all SNPs with MAF >1% may be used. We established informative axes of variation identified via principal component analysis and highlight important features of the genetic structure of diverse European-descent populations, some studied for the first time at this scale. Finally, we investigated the substructure within each of these 15 populations and identified SNPs that help capture hidden stratification. This work can provide information regarding the designing and interpretation of association results in the International Consortia.

## Introduction

Population stratification can be a major cause of concern in genetic association studies. Specifically, imperfect matching between cases and controls can lead to spurious associations, or failure to detect true associations.^1^ Several ways of accounting for hidden population stratification have been proposed (genomic control (GC) correction, adjusting for ancestry-informative principal components (PCs)), but these approaches are only applicable in genome-wide scale data. The GC^2^ approach uses genomic features of the samples to correct for stratification, and thus avoids inflation in the test statistic.^1^ Population stratification may lead to ‘overdispersion' of the statistics used to test for association; by measuring several polymorphisms across the genome, the degree of this overdispersion may be estimated and taken into account. However, GC may not perform well with too few loci, or may overcorrect and lead to a substantial loss in power.^1^ Menozzi *et al*^3^ described the use of PC analysis (PCA) in human genetics in 1978. PCA summarizes high-dimensionality data by capturing the latent variables that best describe a data set, allowing simple visualization of allele frequency differences among populations. It is possible to correlate PCs of the data with meaningful geographic axes. For example, genetic variation in the first two PCs is closely associated with geographic alignment across Europe.^4, 5, 6^ As with GC, PCA may also be used to correct for population stratification when working with a very large number of markers, ideally genome-wide data sets. However, population stratification is much of a concern in replication studies or studies focusing on a smaller number of variants, in which GC or PCs cannot be readily calculated. To circumvent this problem, adjustment for the genotypes of ancestry-informative markers (AIMs) has been proposed as an alternative approach.

Shriver *et al*^7^ proposed that certain markers with distinct frequency differences across populations may be highly informative for assigning ancestry. These markers are referred to as AIMs. A small number of these AIMs may be used to perform population clustering; between 40 and 80 loci, Rosenberg *et al*^8^ demonstrates convergence to five broad continental clusters. Kidd *et al*^9^ used 128 AIMs to characterize samples from 119 populations into 8 broad clusters, which agree with continental boundaries. Precalculated lists of AIMs are available, although these are mostly applicable only to cross-continental studies,^10, 11^ or require a relatively large set of SNPs.^12^

A different way to derive AIMs is to identify SNPs that contribute highly to the significant PCs (PCAIMs), as first discussed by Paschou *et al.*^13^ SNPs that contribute heavily to the underlying axes of variation will be instrumental in clustering samples along population lines; it follows that these SNPs may be used to assign ancestry. A recent study has identified these PCAIMs for samples of North-Central European and Mediterranean origin, and has shown that they may be used to assign sample ancestry.^14^

In this work, we investigated the structure across closely related European populations. We discuss evidence for stratification using PCA and Fst, a measure of genetic distance among samples. Further, we identified lists of AIMs and PCAIMs, which are able to correct for stratification by using a small number of markers.

**Figure 1:**
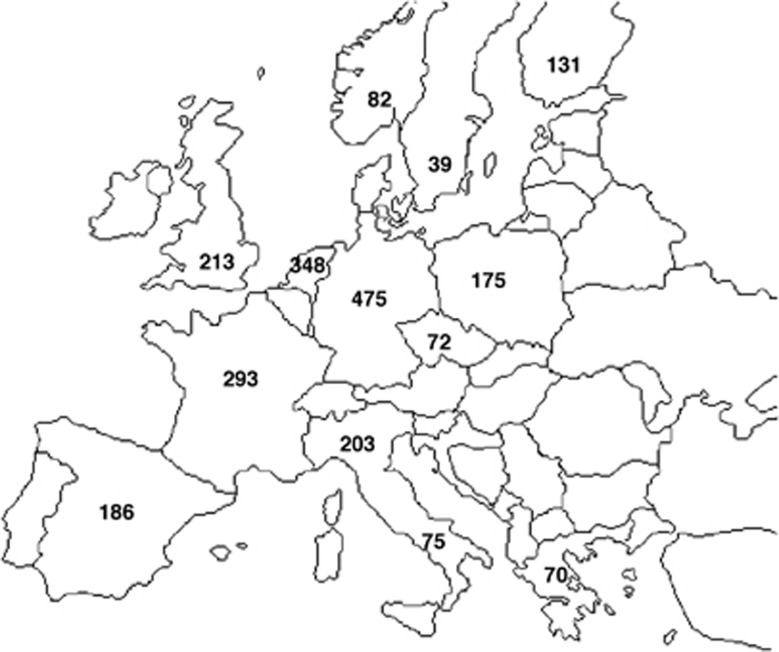
Geographical distribution of samples across Europe.

We investigated population stratification using data taken from the Wellcome Trust Case Control Consortium 3 anorexia nervosa (AN) genome-wide association scan, which includes 2907 cases from 15 different populations of European origin (unpublished data). Thirteen of these are European, and are divided between Scandinavian (Finland, Norway and Sweden), North-Central European (Czech Republic, France, Germany, the Netherlands, Poland and the United Kingdom) and Mediterranean populations (Greece, North Italy, South Italy and Spain). Two further populations of European origin included in this study are United States and Canada. Sample sizes range from 39 (Swedish samples) to 475 (Germany); numbers of samples are shown in Figure 1 and Table 1. Populations were genotyped on the Illumina 670K chip.

We discuss the fine structure within these populations, and identify a set of informative SNPs. We compare different methods of calculating these, and assess their usefulness in assigning samples to populations.

## Materials and Methods

### Sample collection

We used samples that had been collected for an AN GWAS. The samples comprise 15 discovery data sets of European origin. All samples used were female. All samples met the DSM-IV diagnostic criteria for lifetime AN or lifetime ‘eating disorder not otherwise specified', with the exception of the requirement for amenorrhoea. Samples with a lifetime history of bulimia nervosa were also included in the data set.

### Genotyping

All cases were genotyped using the Illumina 660W-Quad arrays (Illumina Inc., San Diego, CA, USA) at the Wellcome Trust Sanger Institute. Quality control was performed individually on each of the 15 case–control subgroups (Supplementary Information).

### PCA

We calculated PCs using the smartpca software (developed at Harvard School of Public Health, Boston, MA, USA).^15^ We identified the top PCs by selecting those components that explained the greatest variance.

We used the Tracy–Widom (TW) statistic to assess the significance of each PC. The TW statistic tests whether the average eigenvector coordinates across all samples within each population differ significantly across components. We found that the first six PCs differ significantly (TW statistic>100, *P*<10^−86^).

### Geographic relevance of PCs

We applied three different tests to calculate the geographic relevance of the PCs. To do this, we first computed the mean eigenvector coordinates of all samples within a population. We then compared these to the centre of genetic variance to the geographic centre. As our samples were obtained from tertiary referral centres, we define ‘Geographic centre' as the geographical midpoint of the country from which the samples were taken. Coordinates were obtained in the same way by Novembre *et al*;^4^ the same coordinates are used here, with the exception of North Italy, which is assigned Verona as its geographic centre.

We then performed the following correlation tests:

1. We used a Spearman's rank correlation coefficient to test for significance of association. Spearman's rank correlations were computed using a standard R package.
2. We applied a Mantel test. This test calculates the correlation between the two distance matrices, and then computes an empirical *P*-value by randomly permuting the rows and columns of one matrix. We performed the Mantel test using the ‘ape' R package^16^ and used 1000 permutations (as recommended).
3. We applied a Procrustes test. This works in the same way as the Mantel test, but is likely to be more sensitive.^17, 18^ We performed the Procrustes test using the ‘vegan' package in R^19^ with 1000 permutations (as recommended).

### FST

Tian *et al*^20^ assign a threshold of Fst=0.001, below which populations may not be said to be genetically distinct.

Fst values were computed using the smartpca software.^15^

To test the correlation between Fst (genetic distance) and geographic distance between population centres, we applied a Mantel test, as for the PCA data.

### AIMs

AIMs are defined as markers that provide information as to the ancestry of a sample. Informativeness describes the amount of information that is imparted by the marker. We use a harmonized data set of 70 samples per population to calculate informativeness. We selected 70 samples per population to avoid any sample-size associated bias in the Informativeness calculation.

Samples were selected at random from all populations; note that Sweden (39 samples) and Canada (54 samples) were omitted owing to small population sizes. The remaining samples were designated as a testing set, to validate AIMs. The Swedish population was set aside to test the ability of AIMs (and PCAIMs) to assign ancestry of samples from a new population.

AIMs were thinned for LD using PLINK.^21, 22^ A threshold of 0.8 was used.

Informativeness was calculated according to Rosenberg *et al*,^8^ using the formula below:


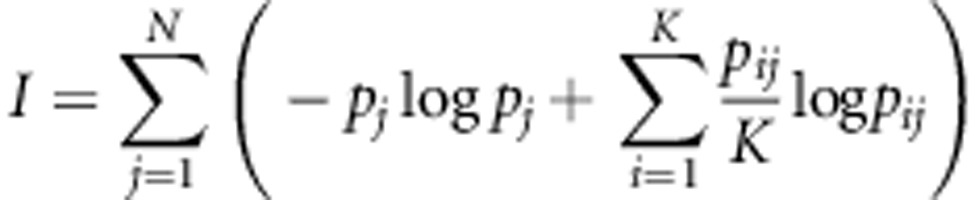


where *p*_*j*_ is the mean frequency of allele *j* over all populations, *p*_*j*_ is the relative frequency of allele *j* in population *i* and *K* is the total number of populations.

### PCAIMs

PCAIMs were selected using a weighting system as outlined by Raaum *et al.*^23^

SNP contributions to each PC were calculated using smartpca.

Contributions of each SNP to each PC were normalized to the maximum weight, so that the SNPs that contributed most to a PC was given a weight of 1. These weights were multiplied by the corresponding eigenvector. To get a rank for each SNP, weights were summed across all PCs.

AIMs were thinned for LD using PLINK.^21, 22^ A threshold of 0.8 was used.

### K-nearest neighbour

K-nearest-neighbour assignments were used to assess how well AIMs and PCAIMs were able to assign a sample to a certain population. (Here, we used *K*=5.). The KNN algorithm identifies the K-nearest genetic neighbours by computing Euclidean distances between samples. We used PLINK to find each sample's K-nearest genetic neighbours, based on only a given number of AIMs. Clustering samples that are ‘closest' together according to a genetic similarity measure, derived by AIMs or PCAIMs, implies that the nearest neighbours share common ancestry with the sample in question. The ancestry of the nearest neighbours was used as a ‘majority vote' to determine the ancestry of the sample.

In cases where the five nearest neighbours did not reach a majority vote, only the four nearest were selected, and a majority vote again taken. If this was still unsuccessful, only the top three were used. If still no majority vote was reached, the sample was classed as ‘unassigned'.

Ancestry was assigned to a sample based on the result of the majority vote. Each sample was considered correctly assigned if the result of the majority vote was either the true ancestry of the sample or a population with a pair-wise Fst<0.001 with the true population.

## Results

### Evidence of structure among populations

**Figure 2:**
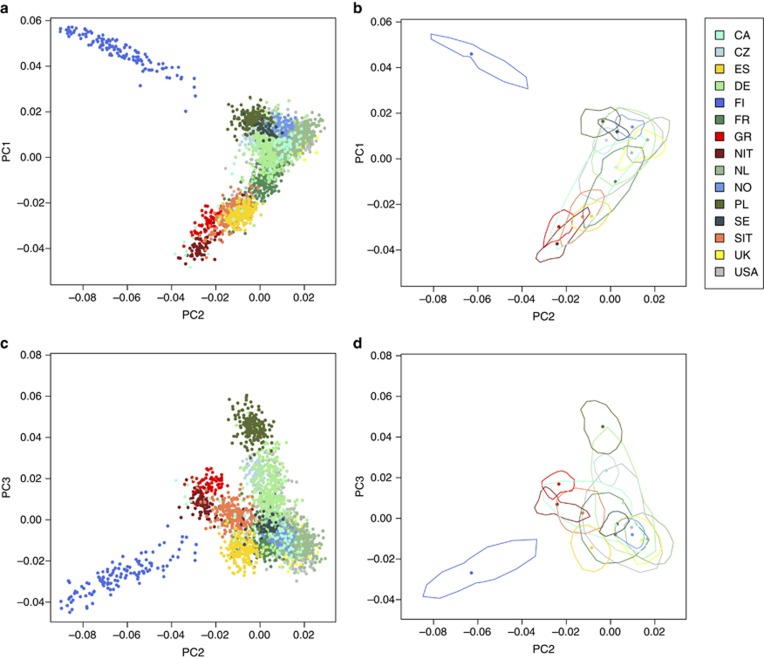
Fine structure between the 15 European populations studied. (**a**) Fine structure across all populations: PC1 *versus* PC2. (**b**) The distribution of samples is shown for each population. Outlying samples (deviating in location by more than 3 SDs from the mean) were excluded. A three-point moving average filter was used to smooth outlines. (**c**) Fine structure across all populations: PC2 *versus* PC3. (**d**) The distribution of samples is shown for each population, calculated as in (**b**). CA, Canada; CZ, Czech Republic; DE, Germany; ES, Spain; FI, Finland; FR, France; GR, Greece; NIT, North Italy; NL, Netherlands; NO, Norway; PL, Poland; SE, Sweden; SIT, South Italy; UK, United Kingdom; USA, United States of America

We performed PCA on the 15 population sets, and plotted the PCs for all populations as shown in Figure 2. The first two PCs accounted for 25.2 and 12.9% of the variation in the data, as shown in Table 2. We used the proportion of variance explained, along with the TW statistic as shown in Table 2, to identify significant PCs.

We tested the geographic relevance of the PCs by calculating the correlation between PC magnitude and latitude and longitude, obtained using the geographic centre of each nation, shown in Supplementary Table 1. Canadian and USA samples were not included in this aspect of the study, owing to the difficulty of assigning meaningful geographic locations. We found that the two top PCs were correlated with perpendicular geographical axes (*ρ*=0.90 for PC1 *versus* latitude, *ρ*=0.59 for PC2 *versus* longitude). After rotation, PC1 aligns north-northwest/south-southeast (NNW/SSE, −11°, *ρ*=0.91). This is remarkably similar to the −16° angle cited by Novembre *et al.*^4^ We see no significant correlation between PC3 and PC4 and geographical axes. We tested for significance between PC locations and geographic centres, and found that this was significant for the first and second PCs (*P*<1e−300 for PC1, *P*=0.036 for PC2, using a Mantel test; *P*=0.001 for PC1, *P*=0.015 for PC2, using a Procrustes test).

Figure 2 presents the first three PCs of the data. Populations form three overlapping subclusters: Finland, central European and Southern or Mediterranean populations. Samples form tight subclusters along population lines, implying that even closely related neighbouring populations are genetically distinct.

USA samples cluster loosely across North-Central European and Scandinavian populations, with some samples clustering with the Mediterranean population. As expected, we see little overlap between Finnish and USA samples. Canadian samples tend to cluster with North-Central European and Scandinavian populations. We performed a PCA using only USA, Canadian, North-Central and Scandinavian populations (therefore removing Mediterranean and Finnish samples), to illustrate this more clearly, as shown in Supplementary Figure 1. This figure confirms the substantial overlap between USA, Canadian and North-Central and Scandinavian populations.

**Figure 3:**
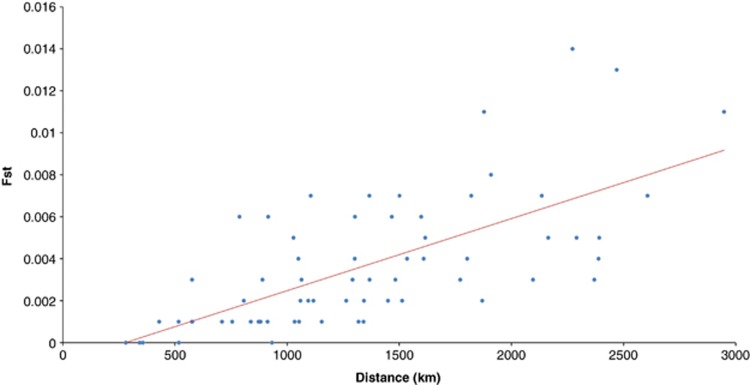
Genetic distance correlates with geographical distance. We computed pair-wise Fst between all populations, and compared this to the geographic distance in kilometres between the midpoints of each population. *R*^2^=0.465.

We calculated genetic distance among populations by means of the Fst statistic (Table 3). Fst correlated well with distance inkilometres between populations (Figure 3) when using the geographic centres of the populations given in Supplementary Table 1. We found a significant correlation between distance in kilometres and Fst (using a Mantel test, *P*<1e−300).

It is clear from Table 3 that a number of pair-wise comparisons between populations show only a very low Fst value. We used a threshold Fst value of 0.001 to identify pairs of populations that are not genetically distinct; this may be owing to recent admixture or shifting of national borders. Pairs of populations that fall below this threshold are shaded in Table 3.

### AIM derivation

We extracted a list of AIMs using Rosenberg's informativeness calculation,^8^ for a harmonized data set of 70 samples per population (for a brief description see the Materials and Methods section). We used 70 samples per population to avoid over-representing populations with larger sample sizes. Populations with fewer than 70 samples were not used to calculate AIMs.

We calculated AIMs using all SNPs with average minor allele frequency (MAF) across all populations >1%. Although it is usual to take 5% as a lower boundary, we find that this risks removing highly informative markers. For example, consider the ‘perfect' marker, which appears in every sample of one population, and not at all in others. For the harmonized set of 13 populations, this marker would have an average MAF of 3.8% across all populations, and would be dismissed under a 5% threshold. We show the top 25 most informative markers in Supplementary Table 2, along with their average MAF. Note that 7 out of these top 25 markers have an average MAF <5%.

One caveat when using AIMs is that populations might not contribute evenly to the choice of markers. A large number of our samples originated from central Europe; although these are classified into distinct populations, we have already shown that some of these populations are very closely related (eg, France and Germany); meanwhile, there were a smaller number of samples from an outlying population (Finland). To ensure that AIMs were chosen evenly to represent all populations, we computed the AIMs using only 12 of 13 populations. We repeated this 13 times, leaving a different population out each time. For each new set of AIMs, we computed the Spearman's rank correlation coefficient with the original list (Table 4). We found an average *ρ*=0.97, although it may be noted that the correlation is slightly lower (*ρ*=0.907) for the set excluding Finland. The high correlations indicate that no single population is over-represented. The lower correlation when excluding the Finnish samples is owing to the greater genetic distance between Finland and other populations.

We use a weighting system as discussed by Raaum *et al*^23^ to select PCAIMs; the top 25 are shown in Supplementary Table 3. We noted that a number of these SNPs fall into clusters (15 of the top 25 cluster on chr. 2, 4 cluster on chr. 15). These locations are associated with geographically restricted positive selection throughout Europe, implying that many of these SNPs may be reflecting the same past event, and may thus not be truly independent. To select SNPs that provide the maximum possible information, we selected only the most informative SNP from each cluster, as shown in Supplementary Table 4.

### Validation of AIMS/PCAIMS

We validated the top AIMs and PCAIMs by testing their ability to assign ancestry to new samples. We used the samples not included in the 70-sample per population harmonized data set; any population with more than 10 samples remaining was included in the validation set.

We used K-nearest-neighbour algorithms to identify possible ancestry of the samples (for a brief description see the Materials and Methods section.

**Figure 4:**
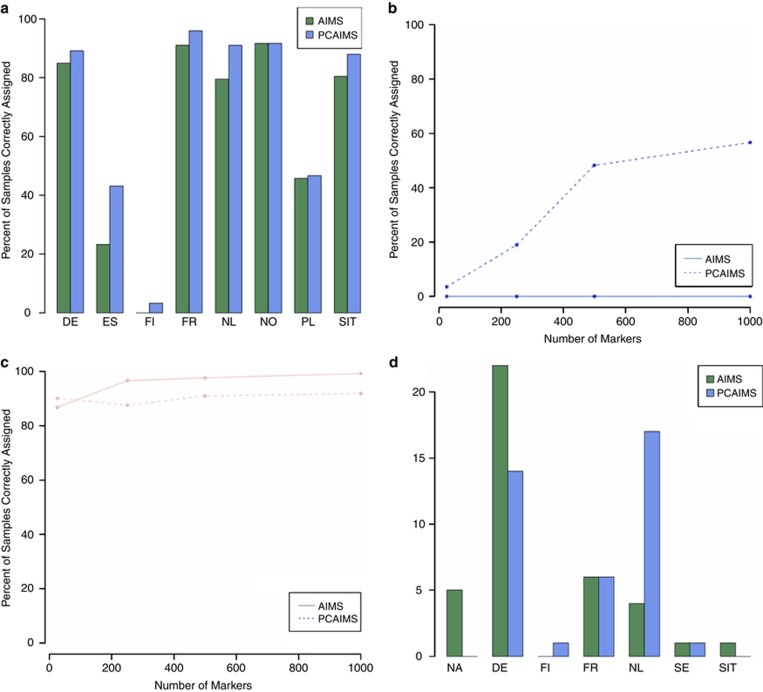
AIMs and PCAIMs are able to predict sample ancestry with high accuracy for most populations, even at small numbers of markers. (**a**) Percent of samples correctly assigned using 25 markers, across all populations. AIMs are shown in green, PCAIMs in blue. (**b**) Assignment of Finnish samples, for varying numbers of markers. AIMs are shown as a solid line and PCAIMs as a dashed line. (**c**) Assignment of German samples, with increasing numbers of markers. (**d**) Assignment of Swedish samples, using 25 markers; AIMs are shown in green and PCAIMs in blue.

Both AIMs and PCAIMs were able to assign ancestry to samples with a high accuracy, even at small numbers of markers. For example, both AIMs and PCAIMs predicted about 90% of the total samples correctly using only 25 markers, although some populations are not predicted well (Spain, Finland and Poland) (Figure 4a).

It may be noted that PCAIMS predict outlying populations better than AIMS. A key example of this is the performance of both sets of markers when predicting Finnish samples (Figure 4b); AIMs predict no samples correctly, even at larger numbers of markers. This failure is due to the way in which AIMs are assigned. We observe high genetic similarity between some central European populations, for example, Czech Republic, France, Germany and Netherlands (as illustrated by low pair-wise Fst values in Table 3). This indicates that a marker that predicts a French sample well will also predict a German sample well. As a sample is considered to be correctly assigned if the final assignment is the original population, or a population with pair-wise Fst <0.001, markers that predict French samples well will also predict German samples well, and will thus increase the number of samples correctly assigned for these populations. In this way, we effectively have 280 samples contributing to ‘Czech/French/German/Dutch' ancestry, as opposed to just 70 Finnish samples. This ties in well with Table 4, as removing any of these four populations still gives a very high correlation of AIMs (*ρ*=0.98). PCAIMs, on the other hand, predict Finnish samples better as they take into account the underlying variation of the data, rather than just the entropy of allelic frequency across samples.

Figure 4c shows the proportion of samples correctly assigned for Dutch populations as a function of the number of markers used. Note that samples are predominantly assigned to neighbouring populations when using PCAIMs, especially Germany and France. A large proportion are assigned correctly, to the Netherlands. When using AIMs, the majority of samples are assigned to Germany, while only a small number are assigned to the Netherlands, and a similar number are left unassigned.

Finally, we considered the assignment of Swedish samples (Figure 4d). This population was not included at all when originally calculating AIMs and PCAIMs; thus, these samples provided an opportunity to see how well a ‘new' population could be assigned using the derived AIMs and PCAIMs. Using PCAIMs, all samples were assigned to geographically close populations, including Germany, the Netherlands and France. We were not able to assign all the samples using AIMs. Further, one sample was assigned to South Italy. All other samples were assigned to geographically close populations using AIMs.

### Substructure within populations

We investigated within-population substructure by performing PCA on each population individually. K-means clustering was then used to assign samples to separate subclusters (Supplementary Figure 2). We found evidence of subclustering in the USA and Canadian populations, and a small number of outliers in the Spanish population. USA samples cluster into three broad groups.

We further investigated substructure within the USA samples by testing nearest-neighbour assignments for the USA samples, using all markers (see Supplementary Material for methods). Each USA sample may then be assigned a ‘nearest' European population, as shown in Supplementary Figure 3. We observe that the majority (74%) of the USA samples cluster with North-Central Europeans, while a further 25% cluster with the Mediterranean populations. We find only a very small number of samples (1%) assigned to Finland, as would be expected.

Substructure within Canadian samples is likely to be due to the large French-Canadian population component. We found that Canadian samples were divided into two groups: a tight cluster and a number of outliers. We plotted Canadian samples alongside French samples, and found that the tight Canadian cluster overlapped the French cluster; outlying samples, on the other hand, did not intersect the French cluster at all (Supplementary Figure 2A).

## Discussion

Population stratification can have a major negative impact on genetic association studies, whether by creating spurious results or by obscuring true associations. This stratification may be corrected using the GC approach, or by adjusting for PCs; however, these methods are only applicable on a genome-wide scale. An alternative approach to this problem is to correct for stratification using AIMs.

We investigated evidence of population stratification across 15 populations of European origin using genome-wide methods such as PCA and Fst. This represents one of the largest studies of this kind, and includes some populations that have not previously been used to assign AIMs (such as Canada and the Czech Republic). Further, these populations are more closely related than those used previously and span a wider geographic range than those seen in recent studies.^14^ For example, we include two Scandinavian populations (Norway and Sweden) and two eastern European populations (Czech and Polish), which are usually clustered into one population. We saw a geographical alignment of our first three PCs. Further, populations cluster along meaningful geographic and cultural lines. We see three broad clusters consisting of Finland, North-Central Europe and Scandinavia, and Mediterranean populations. USA samples cluster largely with North-Central European and Scandinavian samples, with a few clustering with Italian samples, consistent with migratory patterns from Europe to North America.

It appears that Canadian samples cluster closely with French samples; we investigated this in more detail and found that Canadian samples fell into two groups: a tight cluster, which corresponded with the French samples, and a loose cluster, which did not lie close to French samples. This is consistent with some of our samples being of French-Canadian heritage, rather than simply of central European backgrounds.

We also found evidence for substructure within the USA population. We found three broad clusters when performing a PCA plot. We found that most samples cluster with the North-Central European populations (likely to correspond to the largest cluster on our PCA plot), but that there is also a distinct group stemming from Mediterranean populations. This is likely to be due to immigration patterns to the United States. Our third and smallest cluster on the PCA plot is likely to represent a mix of Finnish samples and samples with joint Scandinavian and North-Central European heritage.

We found a correlation between genetic distance, Fst, and the geographic distance between populations. This fits well with the clusters obtained using PCA, and is likely due to admixture between neighbouring populations. In addition, we see very low Fst values between certain pairs of populations, for example, France, Germany and the Netherlands. It is likely that this is due to a lack of significant geographical boundaries in these regions, for example, the Pyrenees or the Alps, and due to shared territories and shifting empire boundaries.

We obtained two lists of AIMs: one list was calculated using Rosenberg's informativeness calculation, and the other using Raaum's PCAIMs. Our initial list of 25 PCAIMs shows that SNPs cluster around three loci, corresponding to lactase and pigmentation-associated loci, *HERC2* and *OCA2*. These genes are classic examples of positively selected genes in European populations, indicating that some of our PCAIMs are picking up high levels of differentiation due to geographically restricted positive selection, rather than due to neutral genetic drift.

Using only a small number of markers, both AIMs and PCAIMs were able to predict sample origin accurately. A key difference between the two sets is the ability to predict ancestry of outlying populations; in this case, PCAIMs outperform AIMs. This is likely to be due to how AIMs and PCAIMs are identified. For example, PCAIMs are chosen to represent the underlying variance of all samples; for our data set, a large part of this variance exists between central European populations and outlying populations (eg, Finland and Spain). As PCAIMs are chosen to explain this variance, even a small number of markers are able to predict outlying populations well.

AIMs, on the other hand, are chosen from markers with a high variance across populations. In this instance, we treat individual populations as independent, and select markers, which explain equally well the difference between all these populations. This is obviously a problem with closely related populations; we can see from PCA graphs that central European populations are in fact not independent; further, we have a much larger number of central European populations than outlying populations, causing a skew towards markers that predict central European populations well.

This difference between the two sets becomes more pronounced when looking at larger numbers of markers. For example, using 500 or 1000 AIMs performs better than PCAIMs in predicting central European nations (ie, in very fine detail), but lag significantly in predicting the ancestry of outlying populations.

We used our lists of markers to assign ancestry to samples from a new population (Sweden), and assessed the ability of our markers to assign ancestry to these samples. Both sets of markers performed well, although PCAIMs perform better than AIMs.

A small proportion of Swedish samples are unassigned using AIMs, whereas all are assigned using PCAIMs. This is likely to be due to the fact that AIMs have been chosen to explain specific differences between a certain set of populations – they may be thought of as discrete measures of differences between populations. PCAIMs, on the other hand, are chosen to represent the continuum of variation. In this respect, we conclude that PCAIMs are better able to explain the ancestry of a new population, as long as it lies on the same continuum.

It is worth bearing in mind the intrinsic limitations of our data set, which consists of clinical samples, obtained by the WTCCC3 for an AN GWAS. Although we have a large number of samples, these have been collected for clinical purposes, rather than for use in population genetics. For this reason, detailed information on ancestry is not always available. Further, samples have been accepted, or excluded, based on clinical relevance and guidelines, rather than based on information about their ancestry. For these reasons, our data may not be as evenly distributed or as well defined as that used in previous population differentiation studies, in which it is usually required that all four grandparents of the sample are also from the region. Further, many anthropological studies focus on rural samples, whereas our samples are statistically more likely to be urban rather than rural. This can also be considered a strength of the study, showing the power of the method to assign ancestry even in a clinically based sample series, which perhaps would not be expected to display the population structure seen in grandparental sampling schemes.

In summary, we derive a set of 25 PCAIMs that can be used to adjust for population stratification within European samples. By genotyping these markers in replication experiments of large-scale genetic association studies, spurious associations arising owing to ancestry differences can be identified and corrected.

## Figures and Tables

**Table 1:** Sample sizes per population

*Population*	*Abbreviation*	*Sample size*
Canada	CA	54
Czech Republic	CZ	72
Finland	FI	131
France	FR	293
Germany	DE	475
Greece	GR	70
North Italy	NIT	203
Netherlands	NL	348
Norway	NO	82
Poland	PL	175
South Italy	SIT	75
Spain	ES	186
Sweden	SE	39
UK	UK	213
USA	USA	491

**Table 2:** Significance of principal components^a^

*Principal component*	% *Variance explained*	*Tracy–Widom statistic*	P-*value*
1	0.14	1333.1	<1E−300
2	0.09	603.3	<1E−300
3	0.07	294.9	<1E−300
4	0.06	121.2	<1E−300
5	0.05	100.9	1.40E−295
6	0.05	43.7	9.79E−86
7	0.05	10.9	3.20E−12
8	0.05	10.0	5.08E−11
9	0.05	10.2	3.24E−11
10	0.05	6.9	5.30E−07

The Tracy–Widom statistic is calculated using the smartpca software package.^15^

a Proportion of variance explained by the top 10 principal components.

**Table 3:** Pair-wise Fst calculated between all populations

	*CZ*	*DE*	*ES*	*FI*	*FR*	*GR*	*NIT*	*NL*	*NO*	*PL*	*SIT*	*UK*
CZ												
DE	0											
ES	0.003	0.002										
FI	0.006	0.007	0.011									
FR	0.001	0.001	0.001	0.008								
GR	0.004	0.004	0.003	0.013	0.003							
NIT	0.005	0.004	0.002	0.014	0.003	0.001						
NL	0.001	0.001	0.003	0.007	0.001	0.005	0.006					
NO	0.002	0.001	0.004	0.006	0.002	0.007	0.007	0.001				
PL	0	0.001	0.005	0.006	0.003	0.006	0.007	0.002	0.003			
SIT	0.003	0.002	0.001	0.011	0.001	0.001	0.001	0.003	0.004	0.004		
UK	0.001	0	0.002	0.007	0	0.005	0.005	0	0.001	0.002	0.002	
USA	0.001	0	0.002	0.007	0	0.004	0.004	0	0.001	0.002	0.002	0

Swedish and Canadian samples are not included here owing to small sample sizes. Population pairs falling below the Fst=0.001 threshold are in pink.

**Table 4:** Correlation between AIMs when calculated using all 13 populations, and when leaving one population out

*Missing population*	*ρ*
CZ	0.9773
DE	0.9810
ES	0.9635
FI	0.9070
FR	0.9795
GR	0.9605
NIT	0.9564
NL	0.9760
NO	0.9713
PL	0.9682
SIT	0.9745
UK	0.9774
USA	0.9771

We calculated AIMs for 13 sets of 12 populations, and computed the Spearman's rank correlation coefficient (*ρ*) in each instance.
