# Supplementary figures and images for: Using ancestry-informative markers to identify fine structure across 15 populations of European origin
Source: Eur J Hum Genet. 2014 Feb 19;22(10):1190–200. doi: 10.1038/ejhg.2014.1 (PMC4169539; doi:10.1038/ejhg.2014.1)

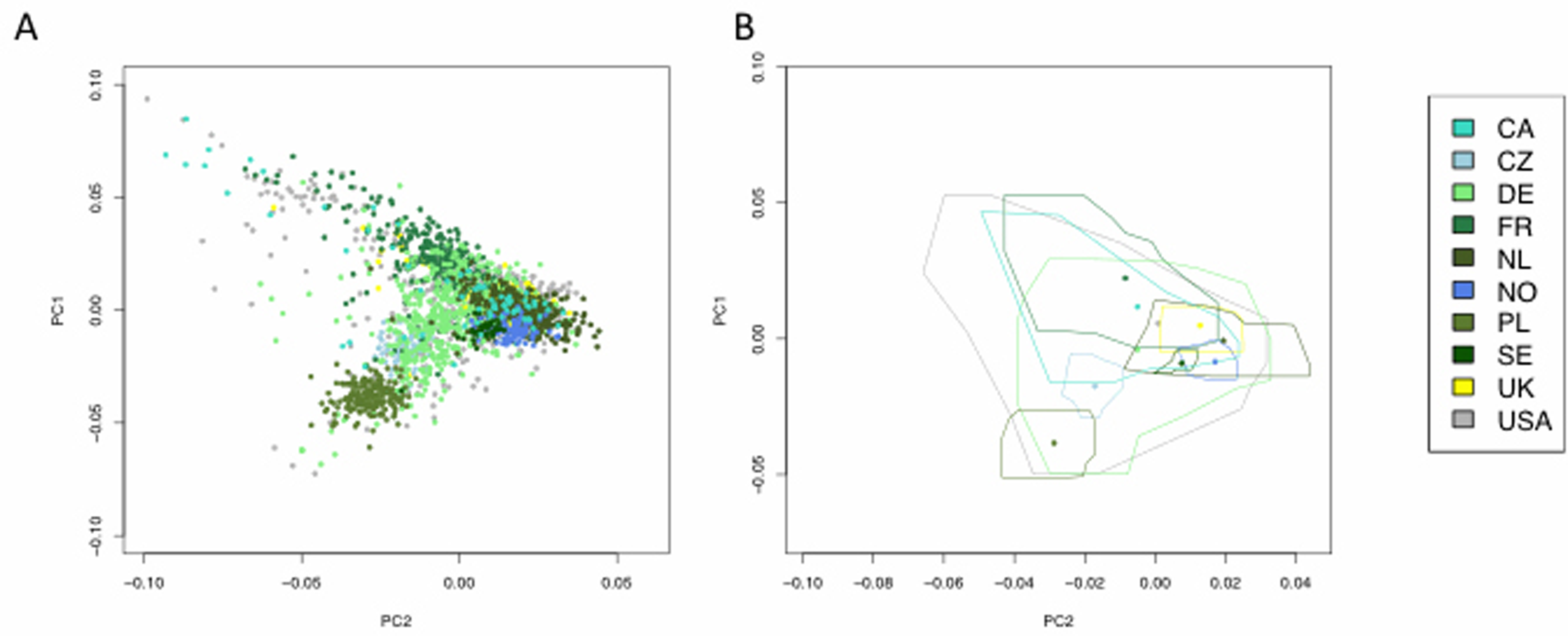

Supplement: Supplementary Figure 1 [file ejhg20141x1.tif]

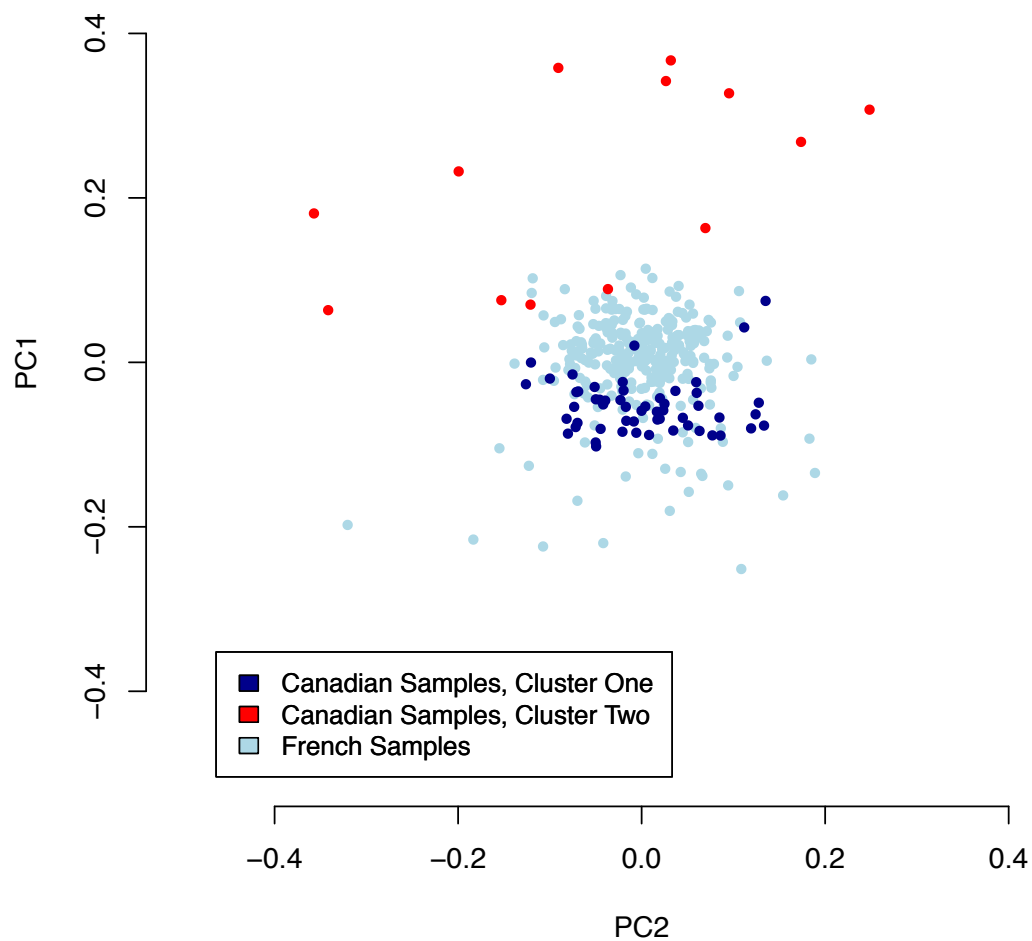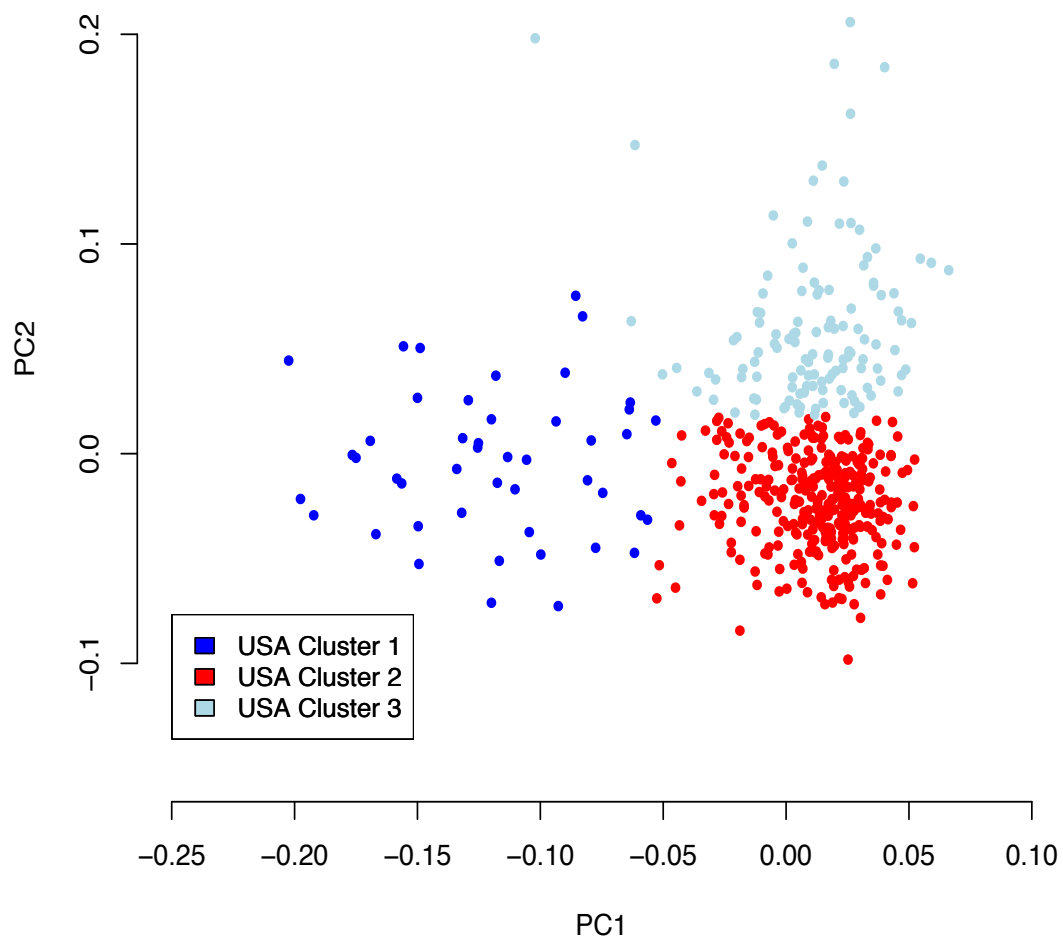

Supplement: Supplementary Figure 2 [file ejhg20141x2.pdf]

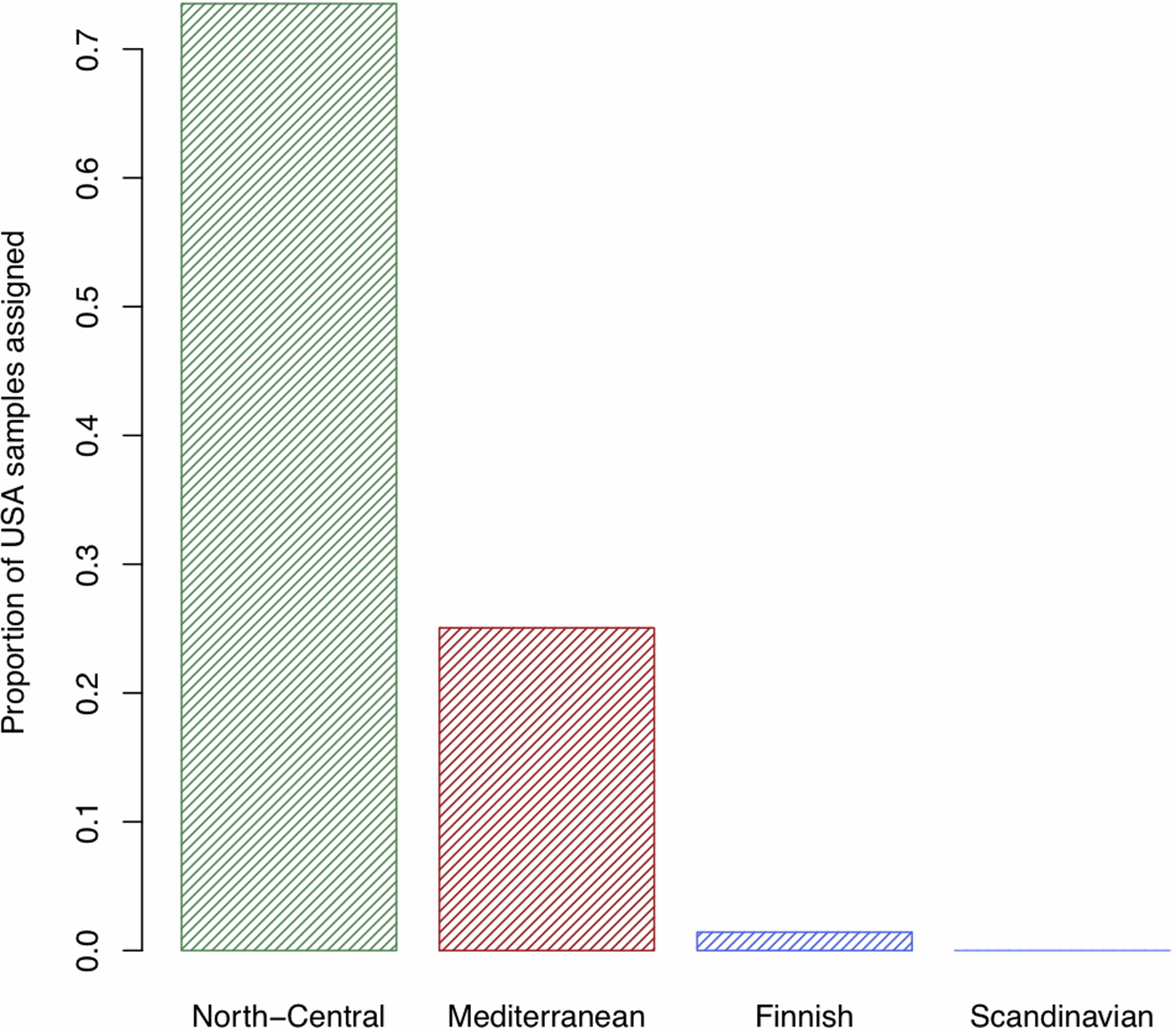

Supplement: Supplementary Figure 3 [file ejhg20141x3.tif]
