# Supplementary material for: Using ancestry-informative markers to identify fine structure across 15 populations of European origin
Source: Eur J Hum Genet. 2014 Feb 19;22(10):1190–200. doi: 10.1038/ejhg.2014.1 (PMC4169539; doi:10.1038/ejhg.2014.1)
Supplement: Supplementary Table 1 [file ejhg20141x4.doc]

Suppl. Table 1. Populations are shown with their geographic centres, obtained from [17], and the latitudes and longitudes of those centres [18]

| Population | City | Latitude | Longitude |
| --- | --- | --- | --- |
| Czech Republic | Cihost | 49.74 | 15.34 |
| Finland | Kajaani | 64.23 | 27.73 |
| France | Paris | 48.52 | 2.2 |
| Germany | Thuringen | 51.01 | 10.85 |
| Greece | Delphi | 38.48 | 22.49 |
| North Italy | Verona | 45.44 | 10.99 |
| South Italy | Naples | 41.54 | 12.29 |
| Netherlands | Utrecht | 52.09 | 5.12 |
| Norway | Ogndalsfjella | 64.02 | 11.53 |
| Poland | Piatek | 52.07 | 19.48 |
| Spain | Madrid | 40.24 | 3.41 |
| Sweden | Flataklocken | 62.38 | 16.3 |
| UK | Slaidburn | 53.98 | -2.45 |
