# Supplementary material for: Using ancestry-informative markers to identify fine structure across 15 populations of European origin
Source: Eur J Hum Genet. 2014 Feb 19;22(10):1190–200. doi: 10.1038/ejhg.2014.1 (PMC4169539; doi:10.1038/ejhg.2014.1)
Supplement: Supplementary Table 2 [file ejhg20141x5.doc]

Supplementary Table 2: The top 25 AIMs. rs IDs and genomic positions refer to build36

| rs ID | Chr | Position | Average MAF |
| --- | --- | --- | --- |
| rs4897268 | 6 | 129042949 | 0.057 |
| rs4788805 | 16 | 70072139 | 0.060 |
| rs17498828 | 4 | 180852651 | 0.053 |
| rs8049837 | 16 | 29996939 | 0.082 |
| rs11861842 | 16 | 30001681 | 0.082 |
| rs17717875 | 2 | 137234086 | 0.062 |
| rs17741142 | 8 | 86403771 | 0.064 |
| rs9912036 | 17 | 11991031 | 0.054 |
| rs1839191 | 2 | 168227203 | 0.048 |
| rs10981818 | 9 | 115348530 | 0.057 |
| rs11653979 | 17 | 47350526 | 0.055 |
| rs6456963 | 6 | 11285675 | 0.056 |
| rs169774 | 16 | 12533127 | 0.049 |
| rs1317636 | 17 | 44906126 | 0.048 |
| rs1604805 | 4 | 21180185 | 0.075 |
| rs6809092 | 3 | 97118452 | 0.061 |
| rs7110543 | 11 | 113029250 | 0.065 |
| rs1877294 | 15 | 75705371 | 0.045 |
| rs9450829 | 6 | 88681156 | 0.051 |
| rs943008 | 6 | 11328158 | 0.053 |
| rs1122905 | 22 | 32324229 | 0.064 |
| rs8124792 | 20 | 52200214 | 0.058 |
| rs2236601 | 1 | 154896061 | 0.044 |
| rs11576066 | 1 | 161150292 | 0.046 |
| rs12464185 | 2 | 107014744 | 0.046 |
