# Supplementary material for: Using ancestry-informative markers to identify fine structure across 15 populations of European origin
Source: Eur J Hum Genet. 2014 Feb 19;22(10):1190–200. doi: 10.1038/ejhg.2014.1 (PMC4169539; doi:10.1038/ejhg.2014.1)
Supplement: Supplementary Table 3 [file ejhg20141x6.doc]

Supplementary Table 3: Top 25 PCAIMs, rs IDs and genomic positions refer to build36

| rs ID | Chr | Position |
| --- | --- | --- |
| rs12913832 | 15 | 28365618 |
| rs7570971 | 2 | 135837906 |
| rs1667394 | 15 | 28530182 |
| rs1561277 | 2 | 136092061 |
| rs10221893 | 2 | 137013606 |
| rs2166480 | 2 | 135637338 |
| rs6723108 | 2 | 135479980 |
| rs1954874 | 2 | 135594699 |
| rs7599054 | 2 | 135540546 |
| rs6430539 | 2 | 135549730 |
| rs6430552 | 2 | 135622808 |
| rs309137 | 2 | 136765951 |
| rs1869829 | 2 | 135877562 |
| rs7495174 | 15 | 28344238 |
| rs6750788 | 2 | 135362981 |
| rs12418058 | 11 | 112908054 |
| rs10164986 | 2 | 135468385 |
| rs7174027 | 15 | 28328765 |
| rs2304933 | 1 | 60102507 |
| rs6599400 | 4 | 1785025 |
| rs6739713 | 2 | 136488978 |
| rs4953922 | 2 | 135402521 |
| rs11117043 | 12 | 86208481 |
| rs209512 | 6 | 53203577 |
| rs842362 | 2 | 135341120 |
