# Supplementary material for: Using ancestry-informative markers to identify fine structure across 15 populations of European origin
Source: Eur J Hum Genet. 2014 Feb 19;22(10):1190–200. doi: 10.1038/ejhg.2014.1 (PMC4169539; doi:10.1038/ejhg.2014.1)
Supplement: Supplementary Table 4 [file ejhg20141x7.doc]

Supplementary Table 4: Top 25 PCAIMs (one per cluster) rs IDs and genomic positions refer to build36

| rs ID | Chr | Position |
| --- | --- | --- |
| rs12913832 | 15 | 28365618 |
| rs7570971 | 2 | 135837906 |
| rs12418058 | 11 | 112908054 |
| rs2304933 | 11 | 59859083 |
| rs2304933 | 1 | 60102507 |
| rs6599400 | 4 | 1785025 |
| rs11117043 | 12 | 86208481 |
| rs209512 | 6 | 53203577 |
| rs842362 | 5 | 105568833 |
| rs1427483 | 7 | 33925764 |
| rs6506664 | 18 | 9399009 |
| rs6840693 | 4 | 74015437 |
| rs2472297 | 15 | 72814933 |
| rs4577828 | 6 | 104402947 |
| rs7320510 | 13 | 29895831 |
| rs10490175 | 2 | 50936258 |
| rs10242595 | 7 | 22740756 |
| rs12451275 | 17 | 12502970 |
| rs3733553 | 4 | 82333527 |
| rs1152653 | 10 | 127749324 |
| rs2815249 | 1 | 215640246 |
| rs16857339 | 3 | 112092405 |
| rs2228375 | 6 | 17783225 |
| rs4279119 | 3 | 67535980 |
| rs7355461 | 2 | 17401317 |
| rs12501555 | 4 | 10912306 |
