# Supplementary material for: Using ancestry-informative markers to identify fine structure across 15 populations of European origin
Source: Eur J Hum Genet. 2014 Feb 19;22(10):1190–200. doi: 10.1038/ejhg.2014.1 (PMC4169539; doi:10.1038/ejhg.2014.1)
Supplement: Supplementary Table 5 [file ejhg20141x8.doc]

| Population | Total number of SNPs | Reason for Exclusion | | | Removed in Total | Number of SNPs Remaining |
| --- | --- | --- | --- | --- | --- | --- |
|  |  | SNPs not called | Failed call rate threshold | Failed Hardy-Weinberg Equilibrium |  |  |
| Canada | 580029 | 262 | 18964 | 539 | 19765 | 560264 |
| Czech | 580029 | 207 | 27082 | 474 | 27763 | 552266 |
| Finland | 580029 | 175 | 20008 | 959 | 21142 | 558887 |
| France | 580029 | 56 | 12965 | 1851 | 14872 | 565157 |
| Germany | 580029 | 139 | 11113 | 2162 | 13414 | 566615 |
| Greece | 580029 | 62 | 24784 | 541 | 25387 | 554642 |
| North Italy | 580029 | 66 | 27955 | 447 | 28468 | 551561 |
| South Italy | 580029 | 202 | 9251 | 1703 | 11156 | 568873 |
| Netherlands | 580029 | 24 | 22973 | 1461 | 24458 | 555571 |
| Norway | 580029 | 227 | 30342 | 564 | 31133 | 548896 |
| Poland | 580029 | 183 | 20469 | 988 | 21640 | 558389 |
| Spain | 580029 | 174 | 13385 | 1258 | 14817 | 565212 |
| Sweden | 580029 | 289 | 11201 | 655 | 12145 | 567884 |
| UK | 580029 | 67 | 22215 | 1186 | 23468 | 556561 |
| USA | 580029 | 49 | 10819 | 2487 | 13355 | 566674 |

Supplementary Table 5: SNP QC Numbers, Autosomes only; Number of SNPs failing each stage of QC, number of SNPs remaining, per population.
