# Supplementary material for: Using ancestry-informative markers to identify fine structure across 15 populations of European origin
Source: Eur J Hum Genet. 2014 Feb 19;22(10):1190–200. doi: 10.1038/ejhg.2014.1 (PMC4169539; doi:10.1038/ejhg.2014.1)
Supplement: Supplementary Table 6 [file ejhg20141x9.doc]

| Population | Total number of SNPs | Reason for Exclusion | | | Removed in Total | Number of SNPs Remaining |
| --- | --- | --- | --- | --- | --- | --- |
|  |  | SNPs not called | Failed call rate threshold | Failed Hardy-Weinberg Equilibrium |  |  |
| Canada | 14194 | 6 | 1184 | 25 | 1215 | 12979 |
| Czech | 14194 | 6 | 1303 | 16 | 1325 | 12869 |
| Finland | 14194 | 5 | 1233 | 22 | 1260 | 12934 |
| France | 14194 | 1 | 1051 | 36 | 1088 | 13106 |
| Germany | 14194 | 2 | 1029 | 40 | 1071 | 13123 |
| Greece | 14194 | 1 | 1292 | 33 | 1326 | 12868 |
| North Italy | 14194 | 1 | 1373 | 17 | 1391 | 12803 |
| South Italy | 14194 | 6 | 954 | 57 | 1017 | 13177 |
| Netherlands | 14194 | 0 | 1251 | 39 | 1290 | 12904 |
| Norway | 14194 | 6 | 1306 | 36 | 1348 | 12846 |
| Poland | 14194 | 4 | 1173 | 29 | 1206 | 12988 |
| Spain | 14194 | 3 | 1053 | 41 | 1097 | 13097 |
| Sweden | 14194 | 6 | 1117 | 45 | 1168 | 13026 |
| UK | 14194 | 2 | 1251 | 39 | 1292 | 12902 |
| USA | 14194 | 0 | 1047 | 65 | 1112 | 13082 |

Supplementary Table 6: SNP QC Numbers, Chromosome X only; Number of SNPs failing each stage of QC, number of SNPs remaining, per population.
