# Supplementary material for: Using ancestry-informative markers to identify fine structure across 15 populations of European origin
Source: Eur J Hum Genet. 2014 Feb 19;22(10):1190–200. doi: 10.1038/ejhg.2014.1 (PMC4169539; doi:10.1038/ejhg.2014.1)
Supplement: Supplementary Figure and Table Legends [file ejhg20141x10.doc]

**SUPPLEMENTARY FIGURE AND TABLE LEGENDS**

Supplementary Figure 1: Fine Structure between populations of North-European and Scandinavian origin.

Abbreviations: CA-Canada; CZ- Czech Republic; DE-Germany; FR-France; GR-Greece; NL-Netherlands; NO-Norway; PL-Poland; SE-Sweden; UK- United Kingdom; USA- United States of America A) Fine Structure across all populations: PC1 vs. PC2. B) The distribution of samples is shown for each population. Outlying samples (deviating in location by more then 3 standard deviations from the mean) were excluded. A three-point moving average filter was used to smooth outlines.

Supplementary Figure 2: A) Population substructure within Canadian samples. Canadian samples form two clusters, shown in dark blue and red. French samples, shown in light blue, align with the first Canadian cluster. B) Population substructure within USA samples. Samples form three clusters, as shown.

**Supplementary Figure 3**: Proportion of USA Samples assigned to North-Central, Mediterranean, Finnish and Scandinavian populations. We computed assignments using a k-nearest neighbour approach, using all markers.

**Suppl. Table 1**: Populations are shown with their geographic centres, obtained from [17], and the latitudes and longitudes of those centres [18]

**Suppl. Table 2**: The top 25 AIMs; rs IDs and genomic positions refer to build36

**Suppl. Table 3**: Top 25 PCAIMs; rs IDs and genomic positions refer to build36

**Suppl. Table 4**: Top 25 PCAIMs (one per cluster); rs IDs and genomic positions refer to build36

**Suppl. Table 5**: SNP QC Numbers, Autosomes only; Number of SNPs failing each stage of QC, number of SNPs remaining, per population.

**Suppl. Table 6**: SNP QC Numbers, Chromosome X only; Number of SNPs failing each stage of QC, number of SNPs remaining, per population.
