# Supplementary material for: Using ancestry-informative markers to identify fine structure across 15 populations of European origin
Source: Eur J Hum Genet. 2014 Feb 19;22(10):1190–200. doi: 10.1038/ejhg.2014.1 (PMC4169539; doi:10.1038/ejhg.2014.1)
Supplement: Supplementary Information [file ejhg20141x11.doc]

**Supplementary Information**

**Data Access**

Data used in the study may be accessed via the European Genome-phenome Archive (www.ebi.ac.uk/ega).

**Sample level QC**

First, all SNPs were converted to the forward strand. Sample call rate was calculated and an exclusion threshold of 99% was used. Cross-checking of sample sex was performed (all samples should be female). A heterozygosity threshold of ± 3 standard deviations (SD) from the mean was used. Multi-dimensional scaling analysis (MDS) was performed and plots were visually inspected. An exclusion list with samples of non-European ancestry was generated. Identity by state and identity by descent (IBS, IBD) statistics were calculated to identify duplicated and related samples. An exclusion threshold of >0.05 was used. All samples failing sample-level QC were removed prior to performing SNP-level QC.

**SNP level QC**

SNP call rate was computed and a list of failing SNPs was generated. An exclusion threshold for SNP call rate of <99% was used regardless of allele frequency.

Deviation from Hardy-Weinberg Equilibrium was calculated and a list of failing SNPs was generated. Exclusion threshold was taken as p<1x10^-4.

SNPs failing these QC steps were removed and all analysis carried out on post-QC datasets.

Numbers of SNPs failing each QC stage are shown in Suppl. Table 5 (Autosomes) and Suppl. Table 6 (Chromosome X).

**Assessing population structure within USA samples**

In order to investigate structure among USA samples, we performed a K nearest neighbour analysis using plink^15,16^, as previously. We used all SNPs to obtain the K nearest neighbours. Again, we used K=5. We used all European populations (ie, all datasets except the USA and Canadian samples) to search for nearest neighbours.

**Minor Allele Frequency (MAF) Spectra**

MAF spectra were computed for each population individually, and across all samples, to provide an average European spectrum, using plink^15,16^. We looked for differences between population and European-wide spectra using a Kolmogorov-Smirnov (KS) test. This test quantifies a distance between two distribution functions (in this case, the distribution function of one population spectrum, compared to the distribution function of the European spectrum). The empirical distance between the two distributions is then used to assess whether the two MAF spectra are significantly different. A deviation from the European spectrum is an indication that fine structure exists among populations. We found a significant difference between individual spectra and the European spectrum (p<2e-16 in all populations).
